# Consensus molecular subtypes classification of colorectal cancer as a predictive factor for chemotherapeutic efficacy against metastatic colorectal cancer

**DOI:** 10.18632/oncotarget.24617

**Published:** 2018-04-10

**Authors:** Akira Okita, Shin Takahashi, Kota Ouchi, Masahiro Inoue, Mika Watanabe, Mareyuki Endo, Hiroshi Honda, Yasuhide Yamada, Chikashi Ishioka

**Affiliations:** ^1^ Department of Clinical Oncology, Institute of Development, Aging and Cancer, Tohoku University, Aoba-ku, Sendai, Japan; ^2^ Department of Medical Oncology, Tohoku University Hospital, Aoba-ku, Sendai, Japan; ^3^ Department of Clinical Oncology, Akita University Graduate School of Medicine, Akita, Japan; ^4^ Department of Pathology, Tohoku University Graduate School of Medicine, Aobaku, Sendai, Japan; ^5^ Department of Pathology, Sendai Kousei Hospital, Aobaku, Sendai, Japan; ^6^ Department of Surgery, Tohoku Rosai Hospital, Aobaku, Sendai, Japan; ^7^ Department of Clinical Oncology, Hamamatsu University School of Medicine, Higashiku, Hamamatsu, Japan; ^8^ Department of Oncology, National Center for Global Health and Medicine, Tokyo, Japan

**Keywords:** consensus molecular subtypes, chemotherapeutic efficacy, predictive biomarkers, colorectal cancer, DNA methylation status

## Abstract

The consensus molecular subtypes (CMS) classification is one of the most robust colorectal cancer (CRC) classifications based on comprehensive gene expression profiles. This study aimed to clarify whether the CMS is a predictive factor for therapeutic effects of standard chemotherapies for metastatic CRC (mCRC). We retrospectively enrolled 193 patients with mCRCs, and using comprehensive gene expression data, classified them into 4 subtypes: CMS1–CMS4. The associations between the subtypes and treatment outcomes were analyzed. Regarding first-line chemotherapy, irinotecan (IRI)-based chemotherapy was significantly superior to oxaliplatin (OX)-based chemotherapy for progression-free survival (PFS; hazard ratio [HR] = 0.31, 95% confidence interval [CI] 0.13–0.64) and overall survival (OS; HR = 0.45, 95% CI 0.19–0.99) in CMS4. Regarding the anti-epidermal growth factor receptor (anti-EGFR) therapy, CMS1 showed particularly worse PFS (HR = 2.50, 95% CI 1.31–4.39) and OS (HR = 4.23, 95% CI 1.83–9.04), and CMS2 showed particularly good PFS (HR = 0.67, 95% CI 0.44–1.01) and OS (HR = 0.49, 95% CI 0.27–0.87) compared with the other subtypes. The biological characteristics of CMS may influence the efficacy of chemotherapy. CMS might be a new predictive factor for the efficacy of chemotherapy against mCRCs.

## INTRODUCTION

Globally, colorectal cancer (CRC) is the third most common cancer and the second most common cause of cancer-related deaths [1]. The median survival time of metastatic colorectal cancer (mCRC) has been reported to be approximately 8 months with palliative treatment [2], which extends to 25.8–31.4 months when standard chemotherapy is administered [3, 4]. Chemotherapy options for mCRC are diverse, and several treatment regimens use cytotoxic agents and molecular-targeted agents either alone or in combination with other drugs [5]. Biomarkers capable of predicting chemotherapeutic efficacy are required for optimal mCRC treatment.

*RAS* mutation is a biomarker for predicting resistance to anti-epidermal growth factor receptor (anti-EGFR) antibodies, and its status is essential for determining the therapeutic indication of anti-EGFR antibodies [6–9]. In addition to *RAS* mutation, it has recently been reported that the primary CRC tumor site (right-sided or left-sided) was found to be associated with the therapeutic effect of anti-EGFR antibodies, and that the primary tumor site has increasingly been used as a biomarker to select the treatment regimen against mCRC [10, 11]. However, it is thought that there exist molecular biological factors related to the primary site and therapeutic effects of anti-EGFR antibodies that have not yet been identified. Ouchi et al. examined 97 CRC samples using genome-wide DNA methylation analysis and reported that highly methylated CRC (HMCC) was resistant to anti-EGFR therapy [12]. They also reported that the predictability for genome-wide DNA methylation status was better than that for primary tumor localization in anti-EGFR therapy. Lee et al. used the CpG island methylator phenotype (CIMP) as a predictive marker for anti-EGFR therapy and reported similar results [13]. However, DNA methylation status in CRC has not yet been established as a predictive biomarker for anti-EGFR therapy because there has been a lack of prospective studies.

Regarding cytotoxic agents, irinotecan (IRI)-based and oxaliplatin (OX)-based regimens are standard chemotherapies for mCRC, although there is no established biomarker to predict the effects of these two drugs. Therefore, it is generally considered that their therapeutic effects are equivalent regardless of which drug regimen precedes the other if both are used [14]. Zhang et al. suggested that the treatment sequence affected the therapeutic effect in CIMP-positive cases [15], although no further validation has been performed. With the diversification of chemotherapy for mCRC, additional biomarkers need to be developed to determine the optimal treatment strategy for each patient.

The consensus molecular subtypes (CMS) of colorectal cancer is a new classification system that integrates six classifications based on the comprehensive gene expression levels of stage I–IV CRCs [16–21]. The CMS classification was created using >4000 samples with 18 data sets and is one of the most robust classifications for CRC [22]. The CMS classification is divided into four subtypes from CMS1 to CMS4, each of which has a characteristic molecular biological background. The subtype has been demonstrated to be a prognostic factor. Although it is expected that therapeutic strategies for CRC based on CMS will be developed, there have been few reports on the association between CMS and chemotherapeutic efficacy. In this study, we retrospectively examined the significance of CMS as a predictive biomarker of chemotherapeutic efficacy for mCRC.

## RESULTS

### CMS classification

Totally, 193 mCRC patients were retrospectively enrolled in this study. Baseline characteristics of all patients are described in Supplementary Table 1. Among all patients, 113 patients showed synchronous metastases and 80 were recurrent cases. This study included two cohorts: Tohoku University Hospital (TUH) cohort (n = 100) and National Cancer Center Hospital (NCCH) cohort (n = 93). Details and statistical comparisons of the two cohorts are presented in Supplementary Tables 1 and 2. Distribution of the treatment group was significantly different between the two cohorts; however, treatment results showed no significant differences between the two cohorts (Supplementary Table 2). As a result of CMS classification for 193 cases, 21 (10.9%), 53 (27.5%), 69 (35.8%), and 50 (25.9%) were classified into CMS1, CMS2, CMS3, and CMS4, respectively. Supplementary Table 3 and Supplementary Figure 1 present the patient characteristics and overall survival (OS) of each subtype, respectively.

### Clinical outcomes of first-line chemotherapy

**Figure 1:**
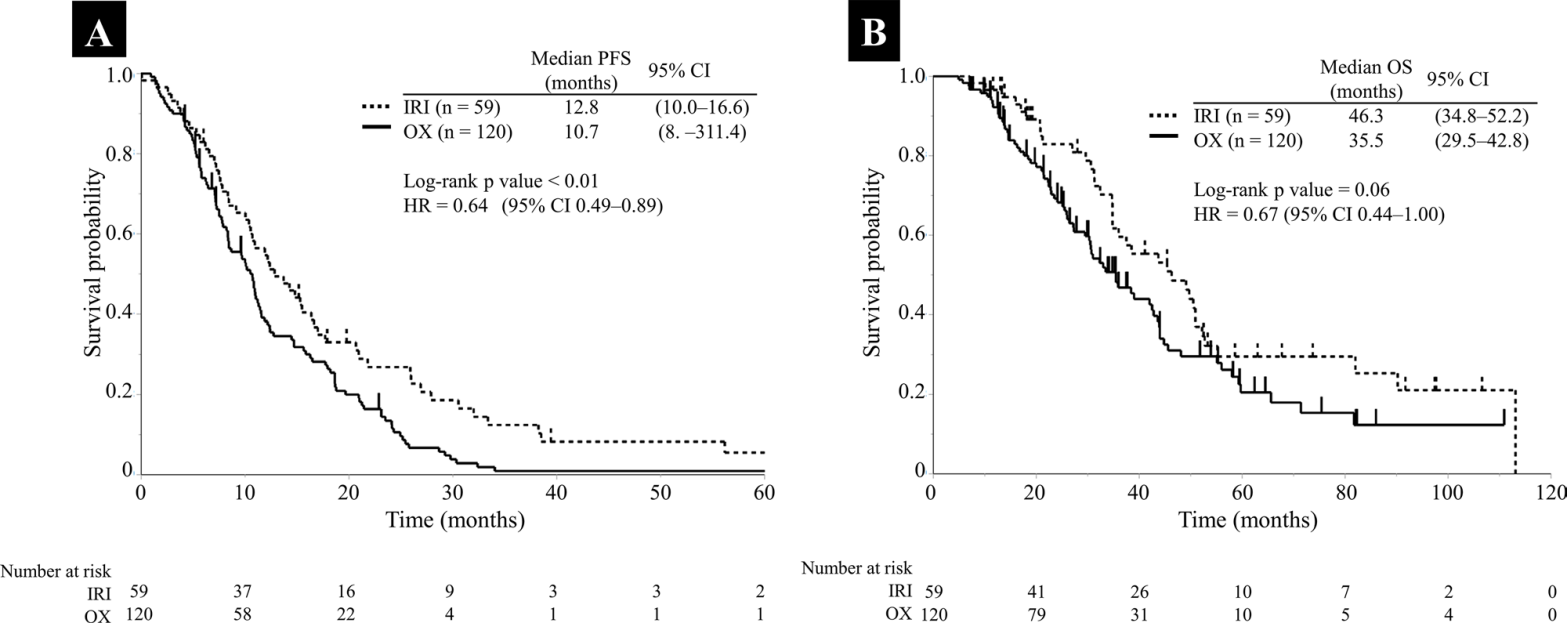
Kaplan–Meier survival curves for PFS and OS in the IRI- (dotted line) and OX-based groups (solid line) **(A)** PFS; **(B)** OS Abbreviations: IRI, irinotecan; OX, oxaliplatin; PFS, progression-free survival; CI, confidence interval; HR, hazard ratio.

In first-line chemotherapy, the patients were classified as the IRI-based group (treated with IRI), OX-based group (treated with OX), and others (Supplementary Table 4). The baseline characteristics of the IRI- and OX-based groups are presented in Table 1. The proportion of patients that received panitumumab combination therapy was significantly higher in the IRI-based group (p < 0.01), and the number of following regimens was significantly higher in the OX-based group (p < 0.01). There were no significant differences in the other baseline characteristics between the groups. Response rate (RR) and disease control rate (DCR) were 70.4% and 98.1% in the IRI-based group and 58.4% and 89.5% in the OX-based group, respectively (Table 2). Both the RR and DCR tended to be higher in the IRI-based group; however, the differences were not significant (p = 0.17 and p = 0.06, respectively). Figure 1 presents the progression-free survival (PFS) and OS in first-line chemotherapy, respectively. The median PFS was significantly better in the IRI-based group than in the OX-based group (12.8 months vs. 10.7 months; log-rank p value < 0.01; hazard ratio [HR] = 0.64, 95% CI 0.49–0.89; Figure 1A). The OS was also better for the IRI-based group than for the OX-based group (46.3 months vs. 35.5 months; log-rank p value = 0.06; HR = 0.67, 95% CI = 0.44–1.00; Figure 1B), although the differences were not significant.

**Table 1:** Baseline characteristics of patients who were received oxaliplatin or irinotecan based regimen as first-line chemotherapy

	All samples					CMS1					CMS2					CMS3					CMS4				
	IRI		OX			IRI		OX			IRI		OX			IRI		OX			IRI		OX		
	n	(%)	n	(%)	p value	n	(%)	n	(%)	p value	n	(%)	n	(%)	p value	n	(%)	n	(%)	p value	n	(%)	n	(%)	p value
Total	59		120			5		16			15		32			26		37			13		35		
Cohort					<0.01					1.00					<0.01					0.15					<0.01
TUH	45	(76.3)	49	(40.8)		2	(40.0)	6	(37.5)		11	(73.3)	9	(28.1)		22	(84.6)	25	(67.6)		10	(76.9)	9	(25.7)	
NCCH	14	(23.7)	71	(59.2)		3	(60.0)	10	(62.5)		4	(26.7)	23	(71.9)		4	(15.4)	12	(32.4)		3	(23.1)	26	(74.3)	
Age, years					0.24					0.62					0.83					0.41					0.71
Median	63		61			52		61			61		61			68		63			62		61		
Range	35–83		29–84			35–73		33–71			49–83		32–75			39–83		29–84			53–70		33–78		
Gender					0.74					0.26					0.52					0.61					1.00
Male	40	(67.8)	77	(64.2)		5	(100)	10	(62.5)		11	(73.3)	19	(59.4)		14	(53.8)	23	(62.2)		10	(76.9)	25	(71.4)	
Female	19	(32.2)	43	(35.8)		0	(0)	6	(37.5)		4	(26.7)	13	(40.6)		12	(46.2)	14	(37.8)		3	(23.1)	10	(28.6)	
Type of metastases					0.05					0.34					0.22					0.60					0.52
Metachronous	18	(30.5)	56	(46.7)		1	(20.0)	8	(50.0)		4	(26.7)	15	(46.9)		8	(30.8)	14	(37.8)		5	(38.5)	19	(54.3)	
Synchronous	41	(69.5)	64	(53.3)		4	(80.0)	8	(50.0)		11	(73.3)	17	(53.1)		18	(69.2)	23	(62.2)		8	(61.5)	16	(45.7)	
Primary tumor location					0.13					1.00					1.00					0.04					0.48
Right colon	24	(40.7)	31	(25.8)		3	(60.0)	8	(50.0)		2	(13.3)	3	(9.4)		16	(61.5)	11	(29.7)		3	(23.1)	9	(25.7)	
Cecum	5		5			1		0			2		1			1		1			1		3		
Ascending	10		17			1		6			0		0			8		7			1		4		
Transverse	9		9			1		2			0		2			7		3			1		2		
Left colon	15	(25.4)	35	(29.2)		0	(0)	2	(12.5)		6	(40.0)	13	(40.6)		4	(15.4)	13	(35.1)		5	(38.5)	7	(20.0)	
Descending	6		4			0		1			2		0			2		2			2		1		
Sigmoid	9		31			0		1			4		13			2		11			3		6		
Rectum	20	(33.9)	54	(45.0)		2	(40.0)	6	(37.5)		7	(46.7)	16	(50.0)		6	(23.1)	13	(35.1)		5	(38.5)	19	(54.3)	
Number of organs with metastasis					0.46					0.33					0.69					0.81					0.22
1	36	(61.0)	68	(56.7)		2	(40.0)	10	(62.5)		9	(60.0)	18	(56.3)		16	(61.5)	22	(59.5)		9	(69.2)	18	(51.4)	
2	22	(37.3)	45	(37.5)		2	(40.0)	5	(31.3)		6	(40.0)	12	(37.5)		10	(38.5)	14	(37.8)		4	(30.8)	14	(40.0)	
≥3	1	(1.1)	7	(5.8)		1	(20.0)	1	(6.3)		0	(0)	2	(6.3)		0	(0)	1	(2.7)		0	(0)	3	(8.6)	
Molecular targeted agents																									
Bevacizumumab	29	(49.2)	58	(48.3)	1.00	3	(60.0)	9	(56.3)	1.00	5	(33.3)	21	(65.6)	0.06	13	(50.0)	11	(29.7)	0.12	8	(61.5)	17	(48.6)	0.52
Cetuximab	0	(0)	0	(0)		0	(0)	0	(0)		0	(0)	0	(0)		0	(0)	0	(0)		0	(0)	0	(0)	
Panitumumab	5	(8.5)	0	(0)	<0.01	1	(20.0)	0	(0)	0.24	3	(20.0)	0	(0)	0.03	1	(3.8)	0	(0)	0.41	0	(0)	0	(0)	
None	25	(42.4)	62	(51.7)	0.27	1	(20.0)	7	(43.8)	0.61	7	(46.7)	11	(34.4)	0.52	12	(46.2)	26	(70.3)	0.07	5	(38.5)	18	(51.4)	0.52
Adjuvant chemotherapy					1.00					0.62					1.00					0.80					1.00
yes	20	(33.9)	42	(35.3)		1	(20.0)	6	(37.5)		4	(26.7)	8	(25.8)		10	(38.5)	16	(43.2)		5	(38.5)	12	(34.3)	
no	39	(66.1)	77	(64.7)		4	(80.0)	10	(62.5)		11	(73.3)	23	(74.2)		16	(61.5)	21	(56.8)		8	(61.5)	23	(65.7)	
unknown	0		1			0		0			0		1			0		0			0		0		
Number of following chemotherapy					<0.01					0.73					0.04					0.04					0.78
0	3	(5.1)	3	(2.5)		1	(20.0)	0	(0)		1	(6.7)	0	(0)		1	(3.8)	1	(2.7)		0	(0)	2	(5.7)	
1	23	(39.0)	21	(17.5)		0	(0)	2	(12.5)		6	(40.0)	5	(15.6)		13	(50.0)	11	(29.7)		4	(30.8)	3	(8.6)	
2	20	(33.9)	54	(45.0)		3	(60.0)	11	(68.8)		2	(13.3)	9	(28.1)		11	(42.3)	17	(45.9)		4	(30.8)	17	(48.6)	
3	11	(18.6)	28	(23.3)		1	(20.0)	2	(12.5)		6	(40.0)	12	(37.5)		1	(3.8)	4	(10.8)		3	(23.1)	10	(28.6)	
≥4	2	(3.4)	14	(11.7)		0	(0)	1	(6.3)		0	(0)	6	(18.8)		0	(0)	4	(10.8)		2	(15.4)	3	(8.6)	
*RAS* mutation					0.72					1.00					0.65					0.78					1.00
+	21	(44.7)	41	(40.6)		2	(40.0)	4	(30.7)		1	(8.3)	5	(17.9)		14	(63.6)	17	(56.7)		4	(50.0)	15	(50.0)	
−	26	(55.3)	60	(59.4)		3	(60.0)	9	(69.2)		11	(91.7)	23	(82.1)		8	(36.4)	13	(43.3)		4	(50.0)	15	(50.0)	
NA	12		19			0		3			3		4			4		7			5		5		
*BRAF mutation*					0.76					1.00					1.00					0.07					1.00
+	5	(8.6)	8	(6.8)		2	(40.0)	7	(43.8)		0	(0)	0	(0)		3	(11.5)	0	(0)		0	(0)	1	(3.0)	
−	53	(91.4)	109	(93.2)		3	(60.0)	9	(56.3)		15	(100)	31	(100)		23	(88.5)	37	(100)		12	(100)	32	(97.0)	
NA	1		3										1								1		2		
DNA methylation status					0.32					0.29					1.00					1.00					1.00
HMCC	6	(27.3)	26	(40.0)		1	(50.0)	10	(90.9)		1	(10.0)	3	(13.6)		3	(50.0)	6	(40.0)		1	(25.0)	7	(41.2)	
LMCC	16	(72.7)	39	(60.0)		1	(50.0)	1	(9.1)		9	(90.0)	19	(86.4)		3	(50.0)	9	(60.0)		3	(75.0)	10	(58.8)	
NA	37		55			3		5			5		10			20		22			9		18		

Abbreviations: TUH = Tohoku University Hospital; NCCH = National Cancer Center Hospital; IRI = irinotecan based group; OX = oxaliplatin based group; CMS = consensus molecular subtype; HMCC = highly methylated colorectal cancer; LMCC = low methylated colorectal cancer; NA = not available.

**Table 2:** Objective response of first-line chemotherapy

	All					CMS 1					CMS 2					CMS 3					CMS 4				
	IRI		OX			IRI		OX			IRI		OX			IRI		OX			IRI		OX		
	n	(%)	n	(%)	p value	n	(%)	n	(%)	p value	n	(%)	n	(%)	p value	n	(%)	n	(%)	p value	n	(%)	n	(%)	p value
CR	4	(7.4)	4	(3.5)		1	(20)	2	(15.4)		1	(7.1)	1	(3.1)		0	(0)	0	(0)		2	(20)	1	(2.9)	
PR	34	(63)	62	(54.9)		2	(40)	5	(38.5)		9	(64.3)	22	(68.8)		17	(68)	16	(47.1)		6	(60)	19	(55.9)	
SD	15	(27.8)	35	(31)		2	(40)	3	(23.1)		4	(28.6)	8	(25)		7	(28)	11	(32.4)		2	(20)	13	(38.2)	
PD	1	(1.9)	12	(10.6)		0	(0)	3	(23.1)		0	(0)	1	(3.1)		1	(4)	7	(20.6)		0	(0)	1	(2.9)	
NE	5		7			0		3			1		0			1		3			3		1		
RR		(70.4)		(58.4)	0.17		(60.0)		(53.8)	1.00		(71.4)		(71.9)	1.00		(68.0)		(47.1)	0.12		(80.0)		(58.8)	0.28
DCR		(98.1)		(89.4)	0.06		(100)		(76.9)	0.52		(100)		(96.9)	1.00		(96.0)		(79.4)	0.12		(100)		(97.1)	1.00

Abbreviations: CMS = consensus molecular subtype; IRI = irintecan based group; OX = oxaliplatin based group; CR = complete response; PR = partial response; SD = stable disease; PD = progressive disease; NE = not evaluable; RR : response rate, DCR : disease control rate.

**Figure 2:**
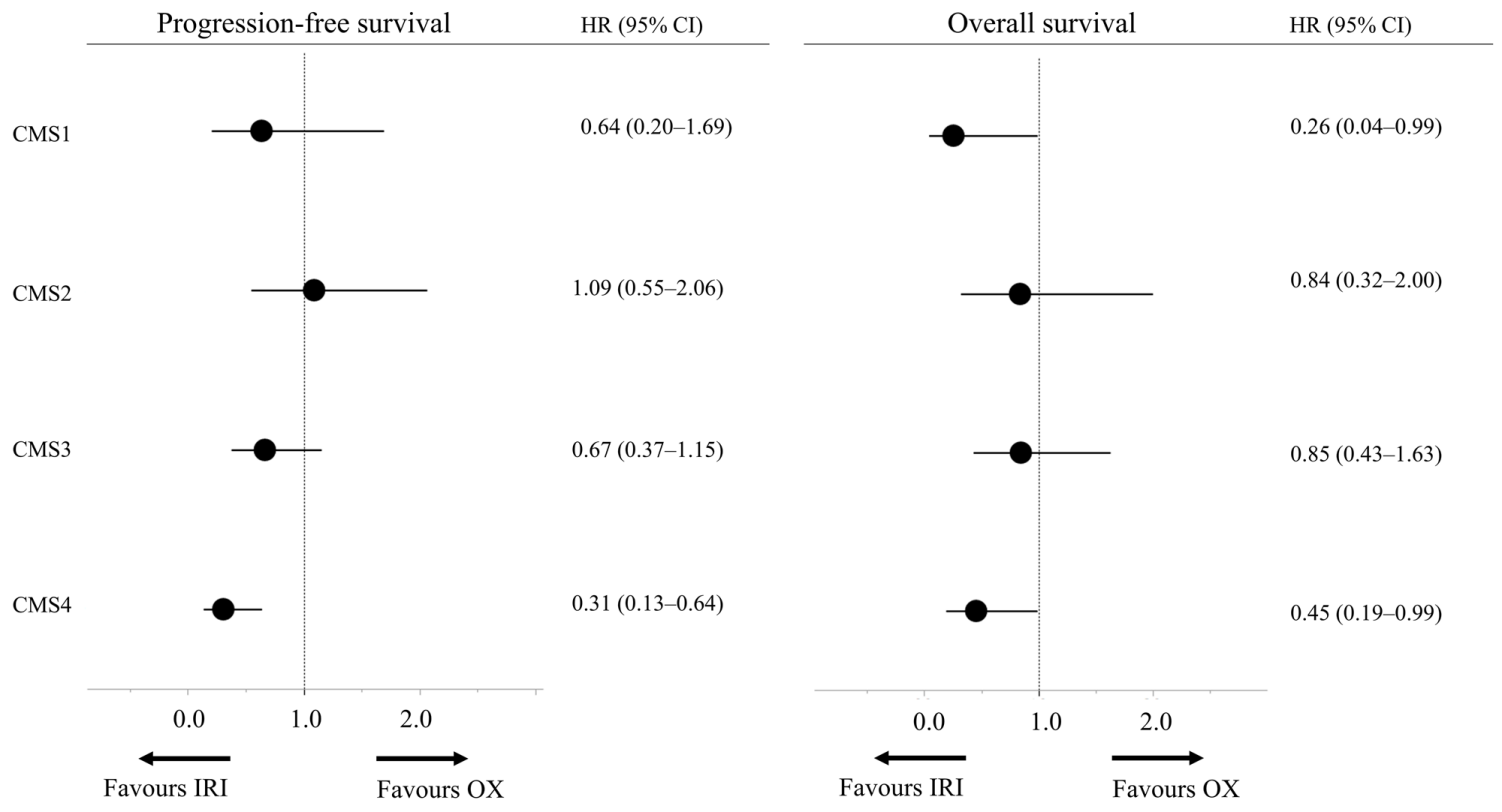
Subgroup analysis based on consensus molecular subtypes Abbreviations: IRI, irinotecan; OX, oxaliplatin; OS, overall survival; CI, confidence interval; HR, hazard ratio.

Next, we also compared the baseline characteristics and clinical outcomes between both treatment groups for each subtype. Only few factors showed significant differences in baseline characteristics (Table 1). For instance, in CMS2, the frequency of panitumumab combination therapy was significantly higher (p = 0.03) in the IRI-based group than in the OX-based group. In terms of clinical outcomes, only in CMS4, both the median PFS and OS were significantly better in the IRI-based group than in the OX-based group (HR = 0.31, 95% CI = 0.13–0.64; HR = 0.45, 95% CI = 0.19–0.99; Figure 2, Supplementary Figures 2 and 3). Regarding objective responses as well, the IRI-based group of CMS4 had the highest RR (Table 2). Overall, the clinical outcomes were better for the IRI-based regimen than for the OX-based regimen in CMS4.

### Gene expression levels of TOP1 and CES2 in CMS4

**Figure 3:**
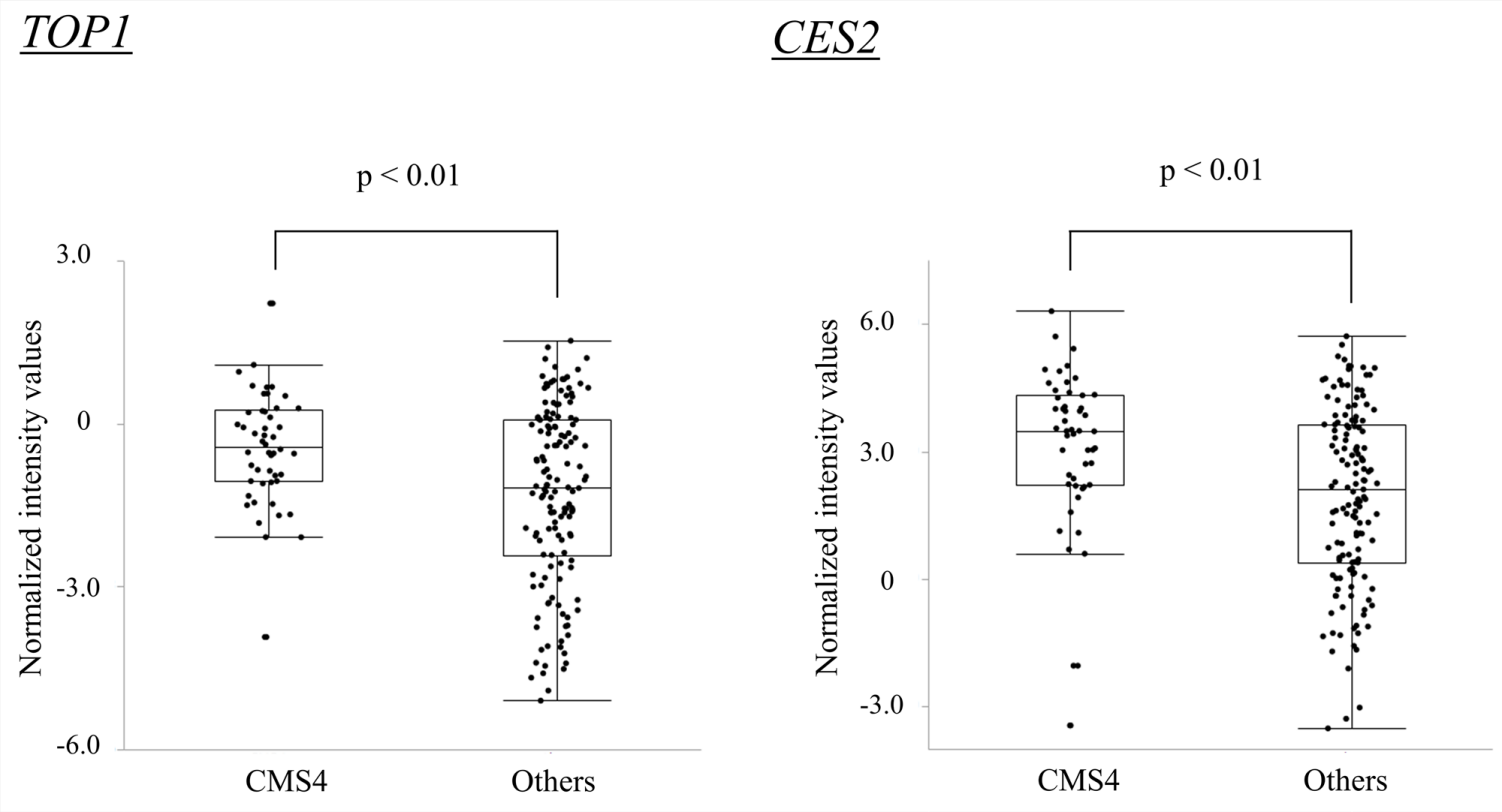
Box-and-whisker plots of the gene expression levels for *TOP1* and *CES2*

Several genes have been suggested to be associated with the therapeutic effects of irinotecan [23–29]. Among the genes previously reported, *TOP1* and *CES2* demonstrated reliable microarray data (high proportion of the cases labeled as “detected”) and the expression levels were significantly higher in CMS4 than in the other subtypes. (Wilcoxon rank-sum test, p < 0.01 and p < 0.01, respectively; Figure 3).

### Clinical outcomes of anti-EGFR therapy

**Figure 4:**
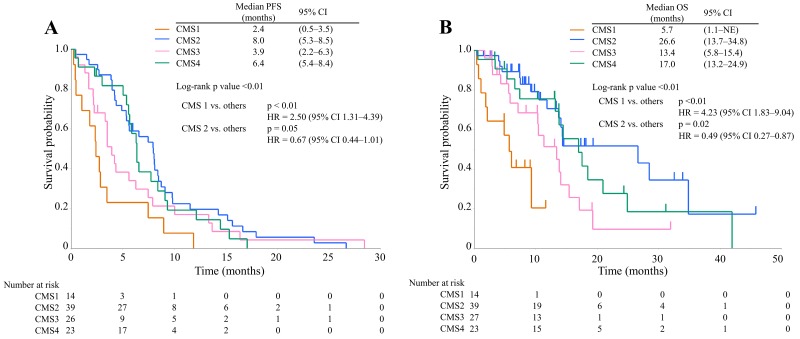
Kaplan–Meier survival curves of anti-EGFR therapy in CMS1 (orange line), CMS2 (blue line), CMS3 (pink line), and CMS4 (green line) **(A)** Progression-free survival time; **(B)** Overall survival time Abbreviations: PFS, progression-free survival; OS, overall survival; CI, confidence interval; HR, hazard ratio.

Next, we analyzed the association between CMS and the therapeutic effects of anti-EGFR therapy. Among 193 patients, 103 without *RAS* mutation were treated with anti-EGFR antibodies. There were 14 patients (13.6%) in CMS1, 39 (37.9%) in CMS2, 27 (26.2%) in CMS3, and 23 (22.3%) in CMS4. The baseline characteristics of each subtype in the 103 patients were similar to those in the 193 patients (Table 3). Table 4 presents the objective response of anti-EGFR therapy for each subtype. RR and DCR were 7.7% and 30.8% for CMS1, 46.2% and 92.3% for CMS2, 29.2% and 58.3% for CMS3, and 31.8% and 81.8% for CMS4, respectively. The DCR of CMS1 was significantly lower than that of the other subtypes (p < 0.01). The RR and DCR of CMS2 were significantly higher than those of the other subtypes (p = 0.05 and p < 0.01, respectively). The PFS and OS of anti-EGFR therapy are presented in Figure 4. The PFS of CMS1 was significantly worse than that of the other subtypes (log-rank p value < 0.01; HR = 2.50, 95% CI 1.31–4.39), and the PFS of CMS2 tended to be better than that of the other subtypes (log-rank p value = 0.05; HR = 0.67, 95% CI 0.44–1.01). The OS of CMS1 was significantly worse than that of the other subtypes (log-rank p value < 0.01; HR = 4.23, 95% CI 1.83–9.04), and the OS of CMS2 was significantly better than that of the other subtypes (log-rank p value = 0.02; HR = 0.49, 95% CI 0.27–0.87).

**Table 3:** Baseline characteristics of 103 patients who were received anti-EGFR treatment

	CMS1		CMS2		CMS3		CMS4		p value
	n	(%)	n	(%)	n	(%)	n	(%)	
Total	14	(13.6)	39	(37.9)	27	(26.2)	23	(22.3)	
Age, years									0.19
Median	55		60		63		61		
Range	33–71		32–83		29–79		33–78		
Sex									0.71
Male	10	(71.4)	27	(69.2)	17	(63)	18	(78.3)	
Female	4	(28.6)	12	(30.8)	10	(37)	5	(21.7)	
Type of metastases									0.15
Metachronous	6	(42.9)	12	(30.8)	11	(40.7)	14	(60.9)	
Synchronous	8	(57.1)	27	(69.2)	16	(59.3)	9	(39.1)	
Primary tumor location									<0.01
Right colon	8	(57.1)	3	(7.7)	12	(44.4)	4	(17.4)	
Cecum	0		2		0		1		
Ascending	6		0		5		1		
Transverse	2		1		7		2		
Left colon	2	(14.3)	14	(35.9)	7	(25.9)	6	(26.1)	
Descending	1		1		2		2		
Sigmoid	1		13		5		4		
Rectum	4	(28.6)	22	(56.4)	8	(29.6)	13	(56.5)	
*BRAF* mutation									<0.01
+	9	(64.3)	0	(0)	3	(11.1)	1	(4.8)	
-	5	(35.7)	38	(100)	24	(88.9)	20	(95.2)	
NA	0		1		0		2		
DNA methylation status									<0.01
HMCC	9	(81.8)	4	(12.1)	7	(38.9)	2	(18.2)	
LMCC	2	(18.2)	29	(87.9)	11	(61.1)	9	(81.8)	
NA	3		6		9		12		
Number of organs with metastasis									0.79
1	9	(64.3)	20	(51.3)	13	(48.1)	13	(56.5)	
2	4	(28.6)	16	(41)	12	(44.4)	9	(39.1)	
3	1	(7.1)	3	(7.7)	2	(7.4)	1	(4.3)	
Number of previous regimens									0.53
0	1	(7.1)	3	(7.7)	1	(3.7)	0	(0)	
1	1	(7.1)	4	(10.3)	3	(11.1)	0	(0)	
≥2	12	(85.7)	32	(82.1)	23	(85.2)	23	(100)	
Type of therapy									0.75
combination with irinotecan	9	(64.3)	30	(76.9)	21	(77.8)	18	(78.3)	
monotherapy	5	(35.7)	9	(23.1)	6	(22.2)	5	(21.7)	
Number of following regimens									0.29
0	10	(71.4)	20	(51.3)	20	(74.1)	11	(61.1)	
1	3	(21.4)	15	(38.5)	5	(18.5)	6	(33.3)	
2	1	(7.1)	2	(5.1)	2	(7.4)	0	(0)	
3	0	(0)	2	(5.1)	0	(0)	1	(5.6)	

Abbreviations: CMS = consensus molecular subtype; HMCC = highly methylated colorectal cancer; LMCC = low methylated colorectal cancer; NA = not available.

**Table 4:** Objective response of anti-EGFR treatment

	All		CMS1		CMS2		CMS3		CMS4		p value
	n	(%)	n	(%)	n	(%)	n	(%)	n	(%)	
CR	0	(0)	0	(0)	0	(0)	0	(0)	0	(0)	
PR	33	(33.7)	1	(7.7)	18	(46.2)	7	(29.2)	7	(31.8)	
SD	39	(39.8)	3	(23.1)	18	(46.2)	7	(29.2)	11	(50)	
PD	26	(26.5)	9	(69.2)	3	(7.7)	10	(41.7)	4	(18.2)	
NE	5		1		0		3		1		
RR		(33.7)		(7.7)		(46.2)		(29.2)		(31.8)	0.07
DCR		(73.5)		(30.8)		(92.3)		(58.3)		(81.8)	<0.01

Abbreviations: CMS = consensus molecular subtype; CR = complete response; PR = partial response; SD = stable disease; PD = progressive disease; NE = not evaluated; RR = response rate; DCR = disease control rate.

### Examination of factors contributing to differences in the therapeutic effect of anti-EGFR therapy

To determine the factors contributing to the therapeutic effect of anti-EGFR therapy against *RA*S wild-type mCRC, univariate and multivariate analyses were performed using Cox proportional hazard models with each characteristic factor as an independent variable (Table 5). Univariate analysis revealed the following significant factors for PFS and OS: CMS (HR = 0.40, p < 0.01; HR = 0.24, p < 0.01, respectively), *BRAF* mutation status (HR = 0.27, p < 0.01; HR = 0.20, p < 0.01, respectively), DNA methylation status (HR = 0.20, p < 0.01; HR = 0.19, p < 0.01, respectively), and type of anti-EGFR therapy (HR = 0.43, p = 0.01; HR = 0.39, p = 0.01, respectively). Multivariate analysis revealed DNA methylation status as only a significant independent factor for both PFS (HR = 0.21, p < 0.01) and OS (HR = 0.30, p = 0.04). In multivariate analysis of PFS, the location of the primary tumor was also an important factor, although it was opposite to the result of univariate analysis (Table 5, Supplementary Figure 4).

**Table 5:** Cox regression analysis for PFS and OS of anti-EGFR treatment

Variable	PFS						OS					
	Univariate			Multivariate			Univariate			Multivariate		
	HR	(95% CI)	p value	HR	(95% CI)	p value	HR	(95% CI)	p value	HR	(95% CI)	p value
Age												
(≧65 vs. <65)	0.64	(0.39–1.01)	0.06	0.71	(0.37–1.31)	0.27	0.65	(0.33–1.17)	0.15	0.73	(0.29–1.76)	0.49
Gender												
(Male vs. Female)	0.83	(0.53–1.31)	0.40	0.69	(0.39–1.26)	0.22	1.29	(0.72–2.44)	0.40	1.20	(0.56–2.78)	0.65
Primary tumor location												
(Left and Rectum vs. Right)	0.84	(0.54–1.36)	0.47	1.98	(1.01–3.94)	0.05	0.56	(0.31–1.05)	0.07	0.92	(0.36–2.41)	0.86
Consensus molecular subtype												
(Others vs. CMS1)	0.40	(0.23–0.76)	<0.01	0.57	(0.23–1.47)	0.24	0.24	(0.11–0.55)	<0.01	0.57	(0.15–2.29)	0.42
*BRAF* mutation status												
(Wild vs. Mutant)	0.27	(0.15–0.54)	<0.01	0.44	(0.18–1.17)	0.10	0.20	(0.09–0.48)	<0.01	0.48	(0.13–1.70)	0.25
DNA methylation status												
(LMCC vs. HMCC)	0.20	(0.11–0.37)	<0.01	0.21	(0.10–0.46)	<0.01	0.19	(0.08–0.46)	<0.01	0.30	(0.10–0.94)	0.04
Type of anti-EGFR therapy												
(Combination with IRI vs. Monotherapy)	0.43	(0.27–0.71)	0.01	0.61	(0.33–1.14)	0.12	0.39	(0.21–0.80)	0.01	0.60	(0.24–1.80)	0.32

Abbreviations: EGFR = epidermal growth factor receptor; PFS = progression-free survival; OS = overall survival; HR = hazard ratio; CI = confidence interval; IRI = irinotecan.

## DISCUSSION

In this study, since the classification was performed using the “nearest CMS”, all cases were classified as one of the 4 subtypes CMS1–CMS4. However, in a previous report, 10% cases could not be classified into any subtype, and it was stated that such cases should be separately considered [22]. Additionally, we performed gene expression analysis using the Whole Human Genome 4 × 44 K Microarray even though the performance of “CMS classifier” on the Agilent platform had been reported as being somewhat inferior [22]. Therefore, it was necessary to verify whether the classification performed in this study reflects the characteristics of CMS. The proportion of CMS3 was higher than previously reported. Because it was higher even in “predicted CMS”, which is a more robust subtype classification (Supplementary Table 5), there may be a distinctive distribution of subtypes in ethnic or this cohort. The molecular biological characteristics of each subtype were similar to those previously reported (Supplementary Table 6), and the prognosis in this study (OS) also showed a trend similar to that in the previous study (survival after relapse) [22]. Thus, the CMS classification in this study was considered reasonable. Although the classification by “predicted CMS” may indicate a more robust biological subtype, in considering clinical applications of CMS as a biomarker for selecting optimal treatment, indeterminate subtypes have to be treated as the nearest subtype. Here, we analyzed the association between “nearest CMS” and the therapeutic effect of chemotherapies.

In the first-line chemotherapy analysis, there were almost twice as many patients in the OX-based group (n = 120) than in the IRI-based group (n = 59). The number of the following chemotherapies was significantly higher in the OX-based group. It is thought that the difference was affected by the treatment strategy when using OX. Peripheral sensory neuropathy is a typical cumulative toxicity of OX, and in patients undergoing prolonged treatment with OX, the neuropathy often requires treatment discontinuation. Therefore, in cases of toxic failure with OX, re-introducing OX in the later lines has been established as a therapeutic strategy [30]. In this study, because the proportion of patients who were reintroduced OX in later line was significantly higher for the OX-based group (25.8%) than for the IRI-based group (6.8%) (Fisher’s exact test, p < 0.01), there might have been significant differences in the number of chemotherapies between the two groups.

The IRI-based group showed a better therapeutic effect than the OX-based group; particularly, PFS was significantly longer in the IRI-based group. Although the proportion of patients that underwent anti-EGFR antibodies combination therapy was significantly higher in the IRI-based group than in the OX-based group, the analysis that excluded cases with anti-EGFR antibodies also showed a significant difference in PFS (Supplementary Figure 5). It is also important that the IRI and OX group each contained multiple regimens, each of which was not a completely uniform treatment group. Furthermore, because this study is a retrospective analysis, some bias may have existed. However, only the treatment regimen revealed an association with PFS as observed by multiple univariate analysis (Supplementary Table 7). In several prospective mCRC clinical trials, IRI-based chemotherapy was associated with longer PFS than OX-based chemotherapy, although the difference was not significant [4, 31, 32]. Therefore, our results appear to be similar to those of the trials. When examined for each subtype, PFS and OS were significantly better in the IRI-based group than in the OX-based group for CMS4. IRI, a topoisomerase1 (topo-1) inhibitor, acts as a prodrug of SN-38 and is converted in the body to SN-38 by carboxylesterase (CES) [33]. Gene expression levels of *TOP1* and *CES2* are expected to be predictive biomarkers for response to IRI and have been analyzed in multiple studies [23–25]. Because the expression levels of the two genes were significantly elevated in CMS4, there was no contradiction as to which CMS4 was sensitive to IRI observed in this study. Although the two genes do not provide sufficient explanation, it is possible that comprehensive gene expression levels may be related to the effects of cytotoxic agents. Del Rio et al. also reported that CMS4 was enriched in FOLFIRI responders by analysis of 143 CRCs [34]. As mentioned above, in first-line chemotherapy, the PFS and OS of IRI have been shown to be better than that of OX [4, 31, 35], and a possible explanation is the low dose intensity of OX due to treatment related peripheral neuropathy [4]. However, our results suggest that the longer PFS of IRI-based regimens may be associated with some biological factors. IRI-based regimens may be more effective as first-line chemotherapy for mCRC, particularly for CMS4, than OX-based regimens.

In the 103 *RAS* wild-type patients treated with anti-EGFR antibodies, the therapeutic effect was particularly low for CMS1. This is consistent with previous first-line chemotherapy reports [36, 37], and CMS is a powerful prognostic factor. In this study, because anti-EGFR antibodies were mostly used after the third regimen, the survival time may more directly reflect anti-EGFR therapeutic effect. To examine the predictive power of CMS for the effect of anti-EGFR therapy on *RAS* wild-type mCRC, univariate and multivariate analyses based on the Cox proportional hazard model for PFS and OS were performed. Although CMS was a significant relevant factor in the univariate analysis, it was insignificant in the multivariate analysis. Contrarily, only DNA methylation status was found to be a significant factor in the multivariate analysis for both PFS and OS. Interestingly, in the multivariate analysis, the location of the primary tumor showed the opposite association to the assumption. The results suggested that genome-wide DNA methylation status is an independent predictive marker for the efficacy of anti-EGFR therapy against *RAS* wild-type mCRC, and in this study, it was a more useful marker than sidedness and CMS. Although identification of subgroups resistant to anti-EGFR therapy is important for considering treatment strategies, identification of subgroups with particularly good response to treatment is also important [38, 39]. From that view point, it is interesting that the therapeutic effect tended to be particularly high for CMS2 patients. This is also consistent with the previous reports [19, 36, 37, 40]. In addition, previous reports suggest that bevacizumab may be more effective for CMS1 than cetuximab [37] and that cetuximab may be more effective for CMS4 than bevacizumab [36]; those are also very important theme.

In this study, we analyzed the effects of CMS on the therapeutic effects of IRI, OX, and anti-EGFR antibodies against mCRC. The limitations of this study were that this was a retrospective analysis, and the number of cases for each subtype was small. However, this is the first study to extensively analyze the associations between CMS and the effects of standard chemotherapies on mCRC. Our study results suggest that IRI is highly effective for CMS4 patients. Biomarkers that can predict the therapeutic effects of cytotoxic agents have not yet been established; however, the present study showed that CMS could be potentially used as a predictive biomarker for the efficacy of an IRI-based regimen. It is expected that the significance of CMS as a predictive biomarker will be verified via prospective or retrospective analyses.

## MATERIALS AND METHODS

### Patients

We retrospectively collected mCRC cases in which formalin-fixed paraffin-embedded primary tumor tissues resected by surgery were available. This cohort comprised 100 patients who received standard chemotherapy at the Tohoku University Hospital from 1998 to 2010 (TUH cohort) and 93 who received standard chemotherapy, including anti-EGFR therapy, at the National Cancer Hospital from 2005 to 2013 (NCCH cohort). This study was approved by the Ethics Committee of the Faculty of Medicine, Tohoku University School of Medicine, and written informed consent was obtained from the targeted patients.

### Gene expression analysis

Gene expression analysis was performed by the Whole Human Genome 4 × 44 K Microarray (Agilent Technologies, Santa Clara, CA, USA) using the same method as that used by Inoue et al. [41]. All gene expression analyses were conducted at our laboratory. Raw data were normalized to a signal value of the 75 percentile of all probes, and the probe sets were filtered by 20–100 percentile. Probes labeled “compromised” were ruled out. All microarray data were available from GSE104645.

### CMS classification using the “CMS classifier”

The CMS classification was performed based on the gene expression profile using the single-sample predictor installed in the R package “CMS classifier” [22]. In this classification method, the similarity of the expression profile to the four subtypes (CMS1 to CMS4) was calculated for each case. Thus, the most similar subtype to the case can be classified “nearest CMS”.

### Gene mutation analysis

Gene mutation analysis was performed using whole exome sequencing and the Luminex Assay (GENOSEARCH™ Mu-PACK™; MBL, Nagoya, Japan) or direct DNA sequencing. Whole exome sequencing was performed in 87 cases. Target enrichment was performed using SureSelect ™ XT Human All Exon V5 + lncRNA (Agilent Technologies, Santa Clara, CA, USA) according to the manufacturer’s protocol on the Illumina HiSeq 2000/2500 platform. For the other 106 cases, direct DNA sequencing of *KRAS* (codons 12 and 13) and *BRAF* (codon 600) was performed using the method described by Inoue et al. [41]. Among the 106 cases, infrequent-*RAS* mutations, including *KRAS* (codons 61 and 146) and *NRAS* (codons 12, 13, and 61), were additionally analyzed in 28 cases using Luminex Assay, and the other 28 cases were analyzed using direct DNA sequencing.

### Genome-wide DNA methylation analysis

We used the genome-wide DNA methylation analysis data reported by Ouchi et al [12]. In summary, genome-wide DNA methylation analysis was performed using an Infinium Human Methylation 450 BeadChip (Illumina, San Diego, CA, USA), and each case was classified as either HMCC or low methylated CRC (LMCC).

### Evaluation of therapeutic effects

Objective responses were evaluated according to the response criteria in solid tumors version 1.0 [42]. The RR was defined as the rate of patients that achieved a complete or partial response, and the DCR was defined as the rate of those that achieved a complete or partial response or a stable disease. OS was defined as the duration from day 1 of each treatment regimen to death. PFS was defined as the duration from day 1 of each treatment regimen to disease progression or death. Treatment discontinuation due to adverse events such as peripheral neuropathy was regarded censored.

### Statistical analysis

The JMP Pro 12 software (SAS, Cary, NC) was used for all statistical analyses. The two-sided Fisher’s exact test and Wilcoxon rank-sum test (or the Kruskal–Wallis test) were used to analyze patient characteristics. Comparison of RR or DCR was performed using the two-sided Fisher’s exact test. Survival curves were constructed using the Kaplan–Meier method with the log-rank test. Hazard ratios for PFS and OS were calculated using the Cox proportional hazards model.

## SUPPLEMENTARY MATERIALS FIGURES AND TABLES

















## Abbreviations

CI: confidence interval
CMS: consensus molecular subtypes
CRC: colorectal cancer
DCR: disease control rate
EGFR: epidermal growth factor receptor
HMCC: highly methylated colorectal cancer
HR: hazard ratio
IRI: irinotecan
IRIS: irinotecan and S-1
mCRC: metastatic colorectal cancer
OS: overall survival
OX: oxaliplatin
PFS: progression-free survival
RR: response rate
SOX: S-1 and oxaliplatin
