# Supplementary material for: Consensus molecular subtypes classification of colorectal cancer as a predictive factor for chemotherapeutic efficacy against metastatic colorectal cancer
Source: Oncotarget. 2018 Apr 10;9(27):18698–711. doi: 10.18632/oncotarget.24617 (PMC5922348; doi:10.18632/oncotarget.24617)
Supplement: Supplementary file 1 [file oncotarget-09-18698-s001.pdf]

Consensus molecular subtypes classification of colorectal cancer as a predictive factor for chemotherapeutic efficacy against metastatic colorectal cancer

SUPPLEMENTARY MATERIALS

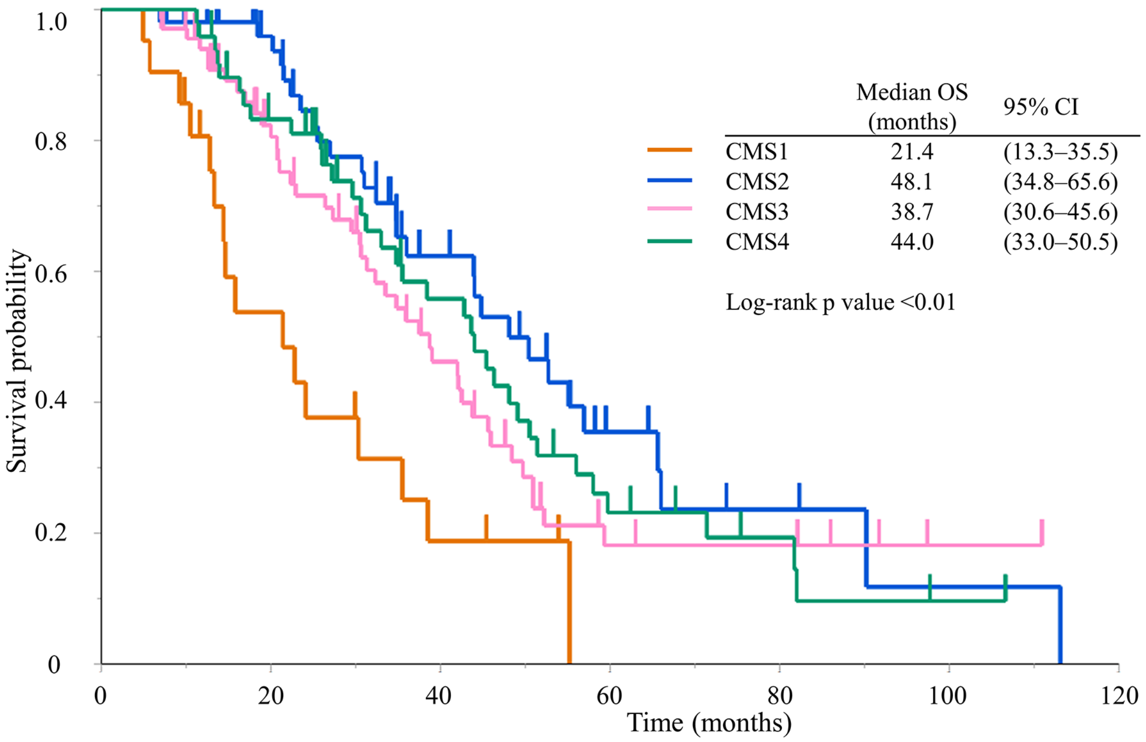

Number at risk							
CMS1	21	10	3	0	0	0	0
CMS2	53	43	21	7	3	1	0
CMS3	69	45	22	6	5	1	0
CMS4	50	38	21	8	4	1	0

Supplementary Figure 1: Kaplan–Meier survival curves for OS of first-line chemotherapy in CMS1 (orange line), CMS2 (blue line), CMS3 (pink line), and CMS4 (green line). Abbreviations: OS, overall survival; CI, confidence interval.

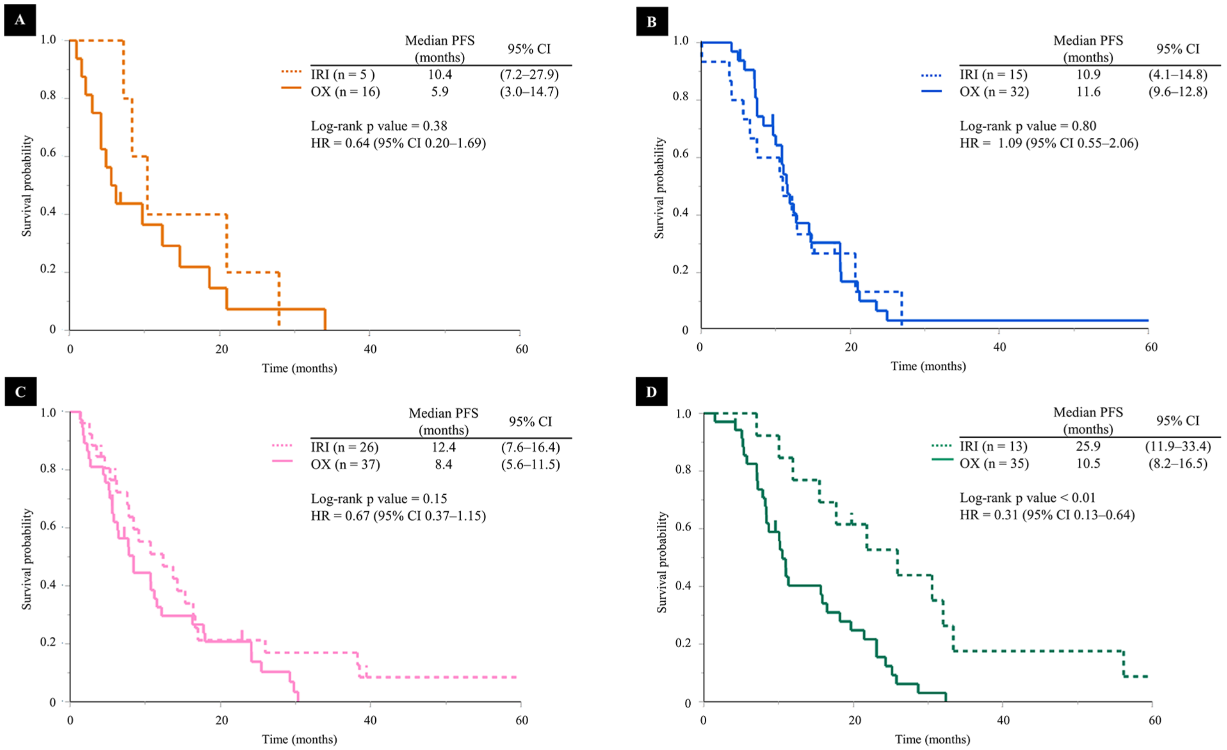

Supplementary Figure 2: Kaplan–Meier survival curves for PFS in the IRI- (dotted line) and OX-based group (solid line). **(A)** Cases of CMS1; **(B)** Cases of CMS2; **(C)** Cases of CMS3; **(D)** Cases of CMS4 Abbreviations: IRI, irinotecan; OX, oxaliplatin; PFS, progression-free survival; CI, confidence interval; HR, hazard ratio.

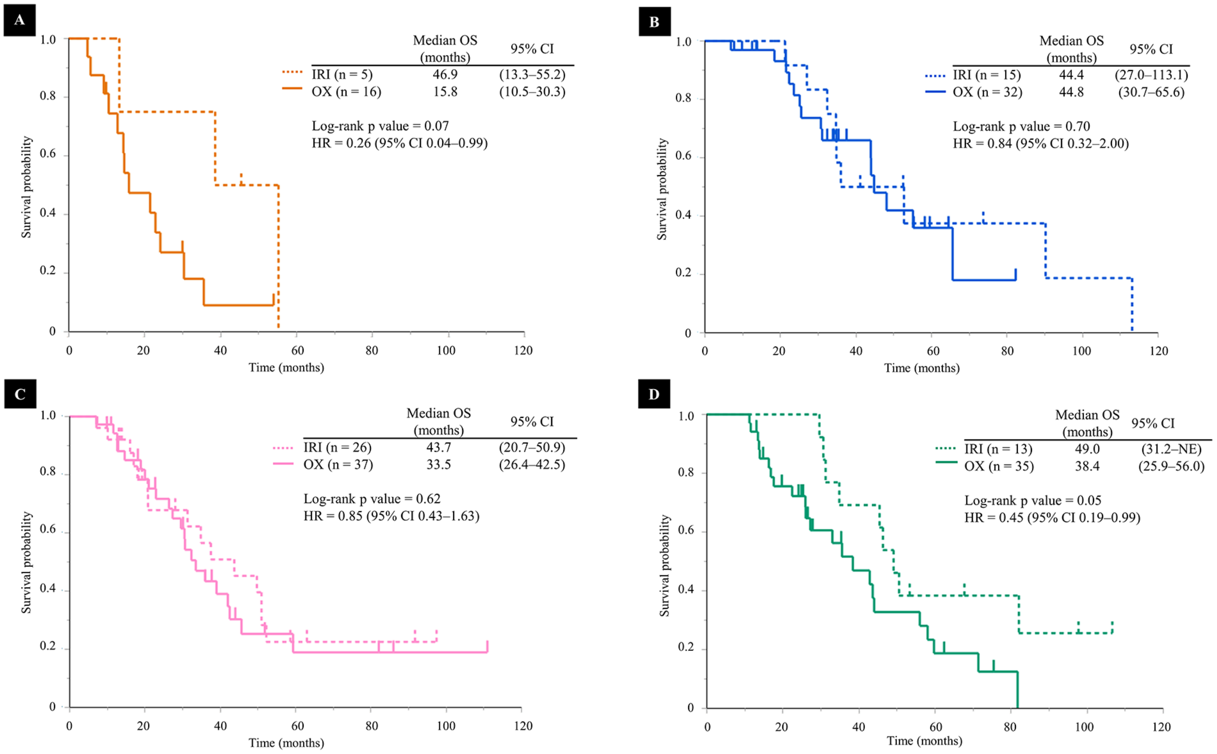

Supplementary Figure 3: Kaplan–Meier survival curves for OS in the IRI- (dotted line) and OX-based group (solid line). **(A)** Cases of CMS1; **(B)** Cases of CMS2; **(C)** Cases of CMS3; **(D)** Cases of CMS4 Abbreviations: IRI, irinotecan; OX, oxaliplatin; PFS, progression-free survival; CI, confidence interval; HR, hazard ratio.

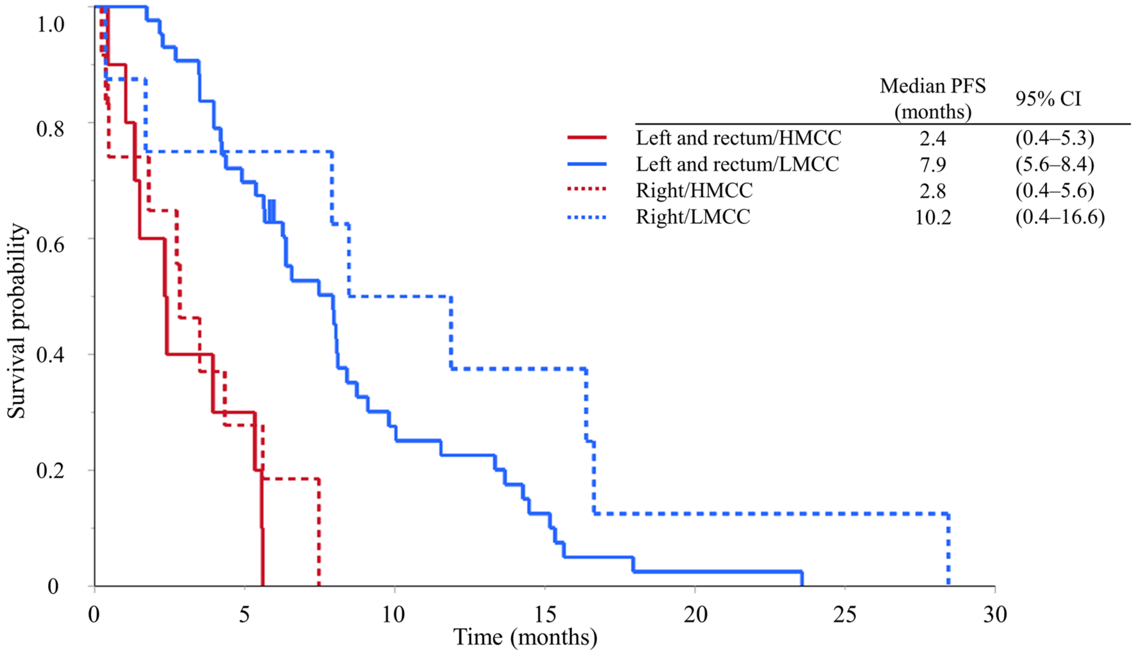

**Number at risk**							
Left and rectum/HMCC	10	3	0	0	0	0	0
Left and rectum/LMCC	43	30	11	5	1	0	0
Right/HMCC	12	3	0	0	0	0	0
Right/LMCC	8	6	4	3	1	1	0

Supplementary Figure 4: Kaplan–Meier survival curves for PFS of anti-EGFR therapy in left colon and rectum tumor (solid lines), right colon tumor (dotted lines), highly methylated colorectal cancer (red lines) and low methylated colorectal cancer (blue lines). Abbreviations: HMCC, highly methylated colorectal cancer; LMCC, low methylated colorectal cancer; PFS, progression-free survival; CI, confidence interval; HR, hazard ratio.

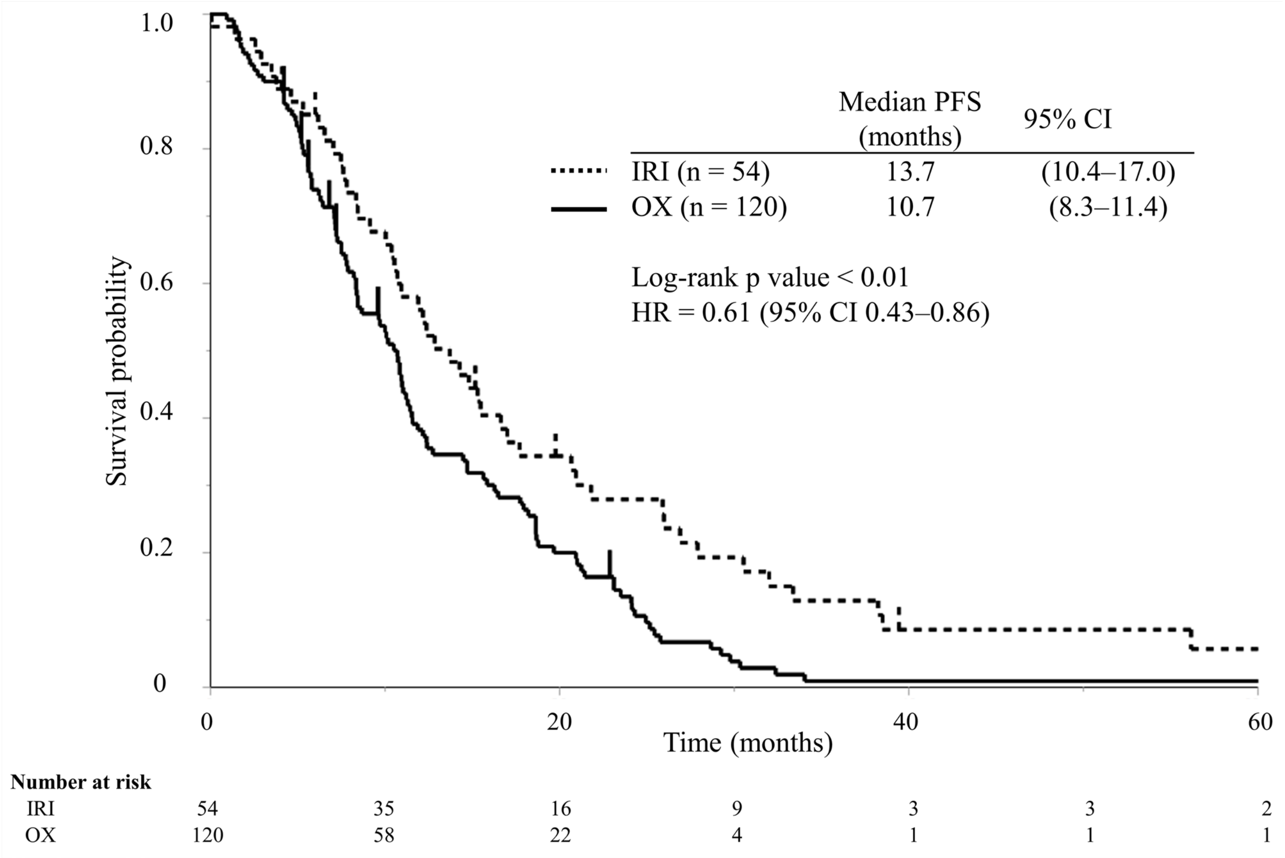

Supplementary Figure 5: Kaplan–Meier survival curves for PFS in the IRI- (dotted line) and OX-based group (solid line), excluding patients who received anti-EGFR antibodies. Abbreviations: IRI, irinotecan; OX, oxaliplatin; PFS, progression-free survival; CI, confidence interval; HR, hazard ratio.

Supplementary Table 1: Detailed information of 193 all patients.

See Supplementary File 1

Supplementary Table 2: Comparison of two cohorts on patient characteristics and clinical outcome.

See Supplementary File 2

Supplementary Table 3: Baseline characteristics of 193 patients.

See Supplementary File 3

Supplementary Table 4:

See Supplementary File 4

Supplementary Table 5: Baseline characteristics of patients classified for "predicted CMS".

See Supplementary File 5

Supplementary Table 6: Comparison of several CMS classifications.

See Supplementary File 6

Supplementary Table 7: Cox regression analysis for PFS of first-line chemotherapy.

See Supplementary File 7
